# Diet Mediate the Impact of Host Habitat on Gut Microbiome and Influence Clinical Indexes by Modulating Gut Microbes and Serum Metabolites

**DOI:** 10.1002/advs.202310068

**Published:** 2024-03-13

**Authors:** Jiguo Zhang, Houbao Qi, Meihui Li, Zhihong Wang, Xiaofang Jia, Tianyong Sun, Shufa Du, Chang Su, Mengfan Zhi, Wenwen Du, Yifei Ouyang, Pingping Wang, Feifei Huang, Hongru Jiang, Li Li, Jing Bai, Yanli Wei, Xiaofan Zhang, Huijun Wang, Bing Zhang, Qiang Feng

**Affiliations:** ^1^ National Institute for Nutrition and Health Chinese Center for Disease Control and Prevention Beijing 100050 China; ^2^ Key Laboratory of Trace Element Nutrition National Health Commission Beijing 100050 China; ^3^ Department of Human Microbiome School and Hospital of Stomatology Cheeloo College of Medicine SD University & SD Key Laboratory of Oral Tissue Regeneration & SD Engineering Laboratory for Dental Materials and Oral Tissue Regeneration Jinan 250012 China; ^4^ Department of Nutrition Gillings School of Global Public Health University of North Carolina at Chapel Hill Chapel Hill NC 27599 USA; ^5^ State key laboratory of microbial technology SD University Qingdao 266237 China

**Keywords:** China population, diet, geography location, gut microbiome, physiological indexes, serum metabolome

## Abstract

The impact of external factors on the human gut microbiota and how gut microbes contribute to human health is an intriguing question. Here, the gut microbiome of 3,224 individuals (496 with serum metabolome) with 109 variables is studied. Multiple analyses reveal that geographic factors explain the greatest variance of the gut microbiome and the similarity of individuals’ gut microbiome is negatively correlated with their geographic distance. Main food components are the most important factors that mediate the impact of host habitats on the gut microbiome. Diet and gut microbes collaboratively contribute to the variation of serum metabolites, and correlate to the increase or decrease of certain clinical indexes. Specifically, systolic blood pressure is lowered by vegetable oil through increasing the abundance of *Blautia* and reducing the serum level of 1‐palmitoyl‐2‐palmitoleoyl‐GPC (16:0/16:1), but it is reduced by fruit intake through increasing the serum level of *Blautia* improved threonate. Besides, aging‐related clinical indexes are also closely correlated with the variation of gut microbes and serum metabolites. In this study, the linkages of geographic locations, diet, the gut microbiome, serum metabolites, and physiological indexes in a Chinese population are characterized. It is proved again that gut microbes and their metabolites are important media for external factors to affect human health.

## Graphical abstract

This population‐level study of Chinese provides a basis for systematically revealing the linkage of geography location, diet, gut microbiome, serum metabolites, and human health. Food is a major mediating factor mediating the impact of host habitat on gut microbiota, and gut microbiota and serum metabolites causally mediate the impact of diet on human health in Chinese population.

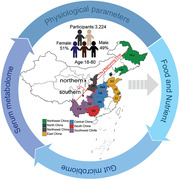

## 1 Introduction

The composition and inter‐individual change of the gut microbiome are modified by host genetics and a complex array of external factors,^[^ ^1^ ^]^ such as habitats, early life exposures, diet, age, ethnicity, and urbanization.^[^ ^2^ ^]^ Among them, host habitats have been reported to be a major factor that affects the composition of the gut microbiota in different countries/regions.^[^ ^2^, ^3^ ^]^ The influence of habitats as an indirect factor on the gut microbiota reflects the accumulative effects of genetic background, daily habits, diets, and multiple environmental factors.^[^ ^4^ ^]^ How habitats affect the gut microbiota has not been elucidated. Since diet is a key factor in shaping the gut microbiota, dietary differences in different regions may be an important factor in revealing the specificity of gut microbes in different habitats.

External factors like diet link gut microbes and serum metabolites with multiple clinical indexes. First, diet exerts an impact on the host metabolome with the help of gut microbes. For example, dietary fiber can be degraded by gut microbes such as *Clostridium*, *Butyrivibrio*, and *Faecalibacterium*, and produce short‐chain fatty acids.^[^ ^5^ ^]^ Fiber‐rich foods (e.g., fruits and vegetables) can also be degraded by microbes from the phyla of *Firmicutes* and raise the circulating level of indole propionate.^[^ ^6^ ^]^ Fructose and sucrose can be transferred to acetate, ethanol, lactate, and succinate by *Blautia producta*.^[^ ^7^ ^]^ Fruit consumption facilitates the level of plasma urolithin B via *Ruminococcus*.^[^ ^8^ ^]^ Second, diet‐ and microbes‐related metabolites can affect human health. For instance, urolithins derived from the polyphenolics of berries and pomegranate fruits by the gut microbiota have a variety of health benefits, including the attenuation of inflammatory signaling, anti‐cancer effects, the repression of lipid accumulation, etc.^[^ ^9^ ^]^ Indoleacrylic acid produced by *Peptostreptococcus* can promote anti‐inflammatory responses.^[^ ^10^ ^]^ One of the studies conducted by the authors showed that *Bacteroides thetaiotaomicron* decreases serum glutamate concentration and inhibits obesity traits such as body mass index, waist circumference, homeostasis model assessment of insulin resistance and triacylglycerol.^[^ ^11^ ^]^ Further, metabolites mediate the gut microbial impact on multiple host clinical indexes revealed by mediation analysis. To take one example, *Ruminococcus sp*. decreases the level of plasma low‐density lipoprotein (LDL) by fostering plasma tyrosol 4‐sulfate, which is a uremic toxin.^[^ ^12^ ^]^ *Ruminococcaceae* UCG‐002 improves type 2 diabetes (T2D) by increasing isolithocholic acids.^[^ ^13^ ^]^ Nevertheless, the knowledge of the connections, especially the causal relationships among diet, the gut microbiome, serum metabolites, and host health is far from adequate. This is due to the difficulties in systematically collecting host external factors and clinical indicators and the high cost of microbiome sequencing in a large population.

To address the above questions, the gut microbiota composition of 3,224 individuals with a comprehensive record of geography, dietary habits, nutrients, and serum metabolites (496 individuals) and physiological status information in China was characterized. In addition, variables significantly correlated with gut microbial variations were characterized, and gut microbiome patterns associated with geography, food, and age in the Chinese population were elucidated. Furthermore, the correlations between diet, the gut microbiota, serum metabolites, and host phenotypes were assessed. This study provides a basis for systematically revealing how external factors affect the gut microbiome, which in turn affects human metabolic and clinical parameters.

## 2 Results

### 2.1 Variables Correlate to the Gut Microbiome in Chinese Population

**Figure 1:**
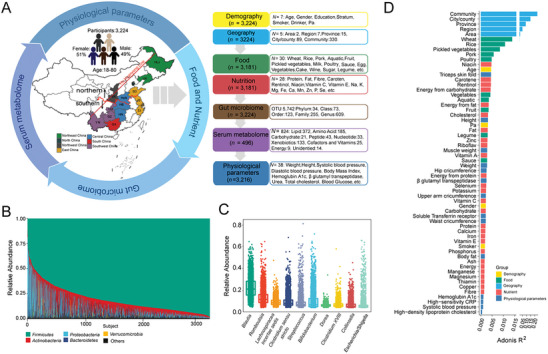
Overview of gut microbiota composition and associated host factors. A) Graphical summary of the cohort and overview of variables (*N* = number of variables collected, *n* = sample size). Background in the map of China is colored according to region classification. The red curve indicates the boundary of two area in this study. The red straight line indicates Hu Huanyong line of China's population boundary. B) The relative abundances of the top five phylum‐level microbiota among individuals (*n* = 3,224). C) The relative abundances of the top ten genera. D) The effect sizes of host factors significantly associated with gut microbial variations were evaluated by PERMANOVA (Adonis) filtered based on FDR < 0.05. The bars are colored according to metadata categories. HLJ, Heilongjiang (*n* = 235); LN, Liaoning (*n* = 141); BJ, Beijing (*n* = 115); SD, Shandong (*n* = 131); JS, Jiangsu (*n* = 146); SH, Shanghai (*n* = 140); ZJ, Zhejinag (*n* = 142); SX, Shaanxi (*n* = 140); HeN, Henan (*n* = 390); HB, Hubei (*n* = 134); HN, Hunan (*n* = 457); CQ (*n* = 148), Chongqing; GZ, Guizhou (*n* = 342); YN, Yunnan (*n* = 130); GX, Guangxi (*n* = 433).

In this study, 3,224 healthy participants (aged 18–80, 51% females) from 330 communities in 89 cities/counties of 15 provinces in the east of the Hu Huanyong Line in China were included (**Figure** **1**A; Tables S1 and S2, Supporting Information). Their feces and fasting blood samples were collected according to standardized procedures (see Experimental Section for details). A total of 109 variables were gathered via questionnaires or physiological examinations and divided into six categories: geography, demography, food, nutrients, physiological, and blood parameters (Table S3, Supporting Information). A microbial catalog of 609 genera from 34 phyla was identified in this cohort by 16S sequencing and taxonomy annotation. Of them, *Firmicutes*, *Actinobacteria*, *Proteobacteria*, *Bacteroidetes*, and *Verrucomicrobia* were the most dominant phyla (Figure 1B). Additionally, *Blautia*, *Romboutsia*, *Bifidobacterium, Clostridium sensu stricto*, *Lachnospiracea incertae sedis*, *Streptococcus*, *Clostridium XVIII*, *Dorea*, *Ruminococcus2* and *Fusicatenibacter* were the ten most abundant genera (Figure 1C; Figure S1A, Supporting Information).

In this study, permutational multivariate analysis of variance (Adonis), analysis of similarities (ANOSIM), multi response permutation procedure (MRPP) and distance‐based redundancy analysis (dbRDA) were applied to analyze the correlations between host variables and the gut microbiome. It was found that 60 variables were significantly correlated with gut bacterial variations across all methods (Table S4, Supporting Information). Adonis analysis showed that geographic factors (community, city/county, province, region, and area) interpreted the greatest variance of the gut microbiome. For example, provincial differences accounted for 17.9% of the microbiome variation. Food intake was another important category affecting gut microbiome composition and accounted for 4.35% of the total (false discovery rate (FDR) < 0.05, Figure 1D). Meanwhile, Spearman's correlation analysis showed that geographic factors were closely related to various foods. For example, provincial differences were significantly associated with the intake of wheat, rice, and pork (Figure S1B, Supporting Information). Geographic information represented by provinces and all individual characteristic information accounted for 24.43% of the variance of the gut microbiome, which suggested that the understanding of the external factors affecting the gut microbiome remains far from enough.

### 2.2 Geographic Locations Affect the Composition of the Gut Microbiota

**Figure 2:**
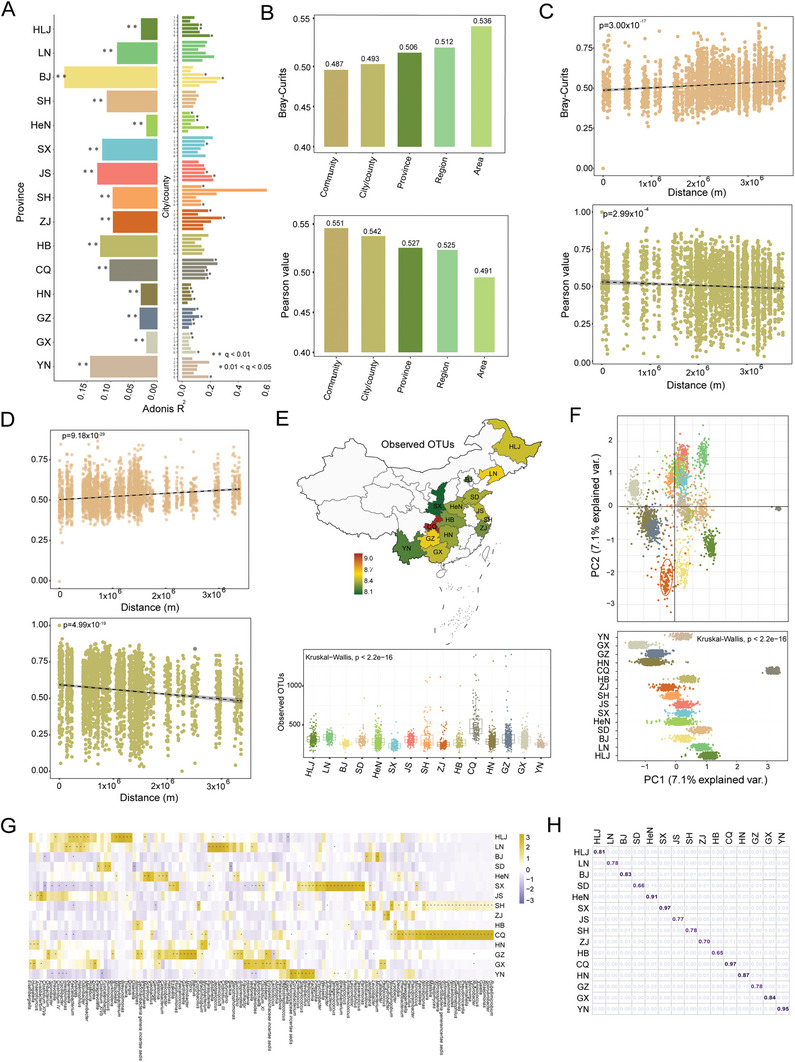
Characterization of geographic‐specific gut microbiota signatures. A) The effect sizes of city/county factor and community factor for gut microbiota variations using Adonis. On the left side of the x‐axis, the effect sizes of city/county factor in each province. On the right side of the x‐axis, the effect sizes of community factor in each city/county (FDR < 0.05). B) The microbiota dissimilarity assessed by Bray‐Curtis distance and microbiota similarity indicated by Pearson coefficient in different geographic ranges. C,D) The linear regression of the relationship between Bray‐Curtis distance or Pearson values and the actual geographic distances from the northernmost resident to the other 3,223 individuals (C) or from the southernmost residents to the other 3223 individuals (D). E) The microbiota α diversity indicated by Observed OTUs in 15 provinces. Background colors in the map of China show the value of local Observed OTUs. F) Linear Discriminant Analysis (LDA) and principal component analysis (PCA) visualizing the beta‐diversity. The values on the PC1 axis in 15 provinces showing downside. The dots were colored according to different province. G) MaAsLin analysis on the microbiome composition and specific genera in 15 provinces (FDR < 0.01). H) Random forest model to determine a person's province location based on province‐specific genera. The number represents the prediction accuracy.

As shown in Figure 1D, geographic locations were most closely related to the gut microbiome, and the finer the division of residential areas was, the greater the impact of residential areas on the gut microbiome would be. To verify this finding, the effects of geographic locations on the gut microbiota were further analyzed. The city/county factor was greatly associated with the gut microbiota profiles in 15 provinces, while the community factor exerted a significant influence on the gut microbiota profiles in 31 cities of 12 provinces (**Figure** **2**A). With the expansion of geographic range, the microbiota dissimilarity assessed by Bray‐Curtis distance increased in different geographic ranges, while the microbiota similarity indicated by the Pearson coefficient decreased (Figure 2B). Moreover, inter‐group microbiota dissimilarity was higher than intra‐group microbiota dissimilarity, while inter‐group microbiota similarity was lower than intra‐group microbiota similarity (Figure S2A,B, Supporting Information). These results suggest that inter‐individual geographic distance is a significant factor influencing the similarity of the gut microbiota.

To further explore the impact of geographic distance on the gut microbiome, differences between HLJ (northernmost province of China) and other provinces were compared. It was discovered that the dissimilarity increased with geographic distance (Figure S2C, Supporting Information). Consistently, the geographic distance between residents in each province and outside the province also showed a significant negative relationship with the Pearson coefficient (Figure S3, Supporting Information). Next, the representatives of the northernmost and southernmost residents were selected to calculate Bray‐Curtis distance and Pearson values for the other 3,223 individuals, respectively. The results also showed that the dissimilarity between representative and other individuals was significantly positively correlated with the increase of geographic distance, regardless of where they were from (p = 3.00 × 10^−17^, 9.18 × 10^−29^, Figure 2C,D). However, the similarity analyzed by the Pearson coefficient was significantly negatively associated with geographic distance (p = 2.99 × 10^−4^, 4.99 × 10^−19^, Figure 2C,D). These results suggest that geographic distance is inversely correlated with the similarity of the gut microbiome.

Next, alpha (α)‐diversity within specific geographic ranges was examined. It was noted that individuals from southern China maintained higher α‐diversity than those from northern China (Figure S4, Supporting Information). At the provincial level, α‐diversity varied among the 15 provinces (Kruskal‐Wallis, *p* < 0.0001), with the highest and lowest in CQ and SX, respectively (Figure 2E; Figure S4, Supporting Information). Concerning beta (β)‐diversity, LDA and PCA showed that all individuals could be clustered by province, region and area, which indicated significant differences on the PC1 axis (Kruskal‐Wallis, *p* < 0.0001) (Figure 2F; Figure S5, Supporting Information). These results suggest that the gut microbiota of individuals living in the same place is more similar.

To identify geography‐specific microbial signatures, multivariate analysis of linear models (MaAsLin) analysis was performed on the microbiome composition of 15 provincial groups at the genus level, respectively. It was observed that each province had at least one high‐abundance genus (Figure 2G). For instance, genera *Methylobacterium*, *Salinicoccus*, and *Fusobacterium* were particularly enriched in HLJ, LN, and JS, respectively, while *Holdemania, Bacillus*, and *Enhydrobacter* were endemic to GX, GZ and ZJ (Figure S6, Supporting Information). Province‐specific bacteria‐based networks showed that the correlations of the gut microbiota also differed across provinces in addition to differences in microbial composition, with SH and CQ maintaining complex bacterial correlations, and SD and ZJ being simpler (Figure S7, Supporting Information). This suggests that geographic locations influence not only gut microbiota composition but also its ecological network. Moreover, a random forest (RF) model was employed to determine that province an individual came from, and an average accuracy of 85% across all provinces was shown (Figure 2H). Of them, the accuracy rate of SX and CQ was as high as 97%, while that of HB was 65%.

Furthermore, MaAsLin was performed on the microbiota composition of seven regional groups at the genus level. Some bacteria were found to be preferentially distributed in certain regions. To be specific, *Lactococcus* and *Fusobacterium*, *Acinetobacter* and *Asaccharobacter*, *Corynebacterium* and *Sphingomonas*, as well as *Streptococcus* and *Blautia* were enriched in South, North, Southwest, and East China, respectively (Figure S6, Supporting Information).

### 2.3 Diet is the Main Factor Mediating Geographic Locations and the Gut Microbiota

**Figure 3:**
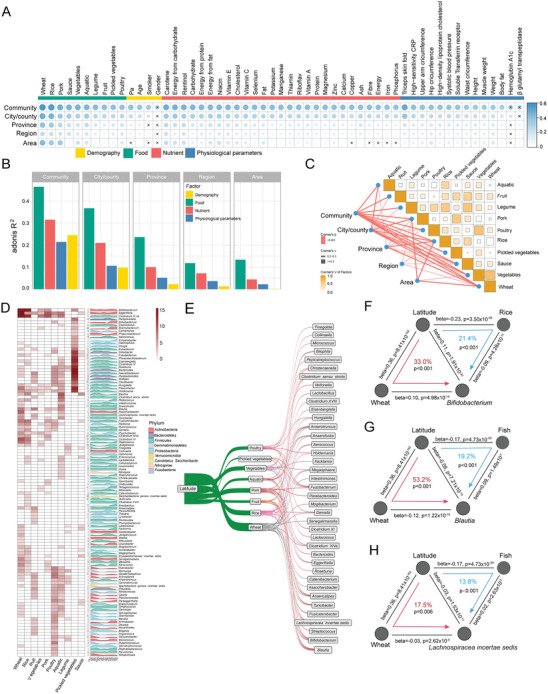
Mediation linkages among geographic location, diet, and the gut microbiome. A,B) Correlations between foods, nutrient, demographic, physiological parameters, and different geographic ranges using Cramer's V (A) and Adonis (B) (*p* < 0.05 and Cramer's V > 0.2). C) Correlation among 10 geography‐associated food analyzed by Cramer's V based on all samples (*n* = 3,181). D) The relationship between geography‐associated food and gut microbiota identified by Boruta (on the left) and the relative abundances of food‐associated microbiota in 15 provinces. The peak plots are colored according to phylum. E) Causal linkages among latitude, food, and the gut microbiota by mediation analysis (*p* < 0.05). F,H) Examples of causal relationships between latitude, food, and the gut microbiota. The gray lines indicate the associations. The red and blue arrowed lines indicate the latitude effects on microbiota mediated by specific food. The beta coefficient and p values are labeled at each edge. The proportions of indirect effect (mediation effect) and mediation p values are labeled at the center of the ring charts.

To identify variables mediating the impact of geographic locations on the gut microbiome, the correlations between food, nutrients, demography, physiological parameters, and different levels of geographic ranges were analyzed using Cramer's V and Adonis. The results consistently showed that all geographic ranges were most strongly related to food variables, particularly 10 different types of food (**Figure** **3**A,C; Figure S8, Supporting Information). The food intake of 3,224 individuals in different geographic regions was analyzed to identify geographic differences in food consumption among the Chinese population. The results revealed significant differences in food intake among 15 provinces, seven regions or two areas (Kruskal–Wallis, *p* < 0.0001, Figure S9, Supporting Information). To be specific, it was noticed that wheat consumption increased with latitude, while rice, pork, and vegetable consumption decreased with latitude (Figure S10, Supporting Information). In addition, individuals from HeN, SX, and SD (northern China) ate more wheat, whereas those from HN, GZ, GX, and YN (southern China) ate more rice, pork, and vegetables (Figure S9, Supporting Information). The relationship between food intake and the gut microbiota was further analyzed. It was found that ten foods were significantly associated with 116 genera (Figure 3D). For instance, wheat intake showed a significant correlation with *Bifidobacterium*, *Blautia*, and *Eggerthella*. These food‐associated genera also exhibited clear geographic differences due to the geographic preference of food. *Bifidobacterium* was enriched in individuals from BJ, SX, and HeN; *Blautia* was enriched in individuals from ZJ and YN; *Eggerthella* was enriched in individuals from YN and GX (Figure 3D; Figure S11, Supporting Information).

Next, mediation analyses were performed on latitude, food intake, and gut microbes, which revealed 76 significant mediation links (p_mediation_ < 0.05, Figure 3E; Table S5, Supporting Information). For example, the intake of wheat and rice could mediate the effect of latitude on *Bifidobacterium* (p_mediation_ < 0.001, Figure 3F). Wheat and fish consumption could mediate the effect of latitude on *Blautia* and *Lachnospiracea incertae sedis* (Figure 3G,H). Regression analysis suggested that wheat intake and the abundance of *Bifidobacterium* were positively correlated with the increase of latitude (β = 0.36, 0.11), while rice and fish intake (β = −0.23, −0.17) and the abundance of *Blautia* and *Lachnospiracea incertae sedis* (β = −0.08, −0.03) were negatively associated with it (β = 0.14, 0.14, 0.04). This indicated that individuals from higher latitudes (northern China) consumed more wheat and less rice and fish, which may be the reason for maintaining more *Bifidobacterium* and less *Blautia* and *Lachnospiracea incertae sedis* (Figure S11, Supporting Information).

### 2.4 The Gut Microbiota Mediates the Impact of Food Consumption on Serum Metabolites

**Figure 4:**
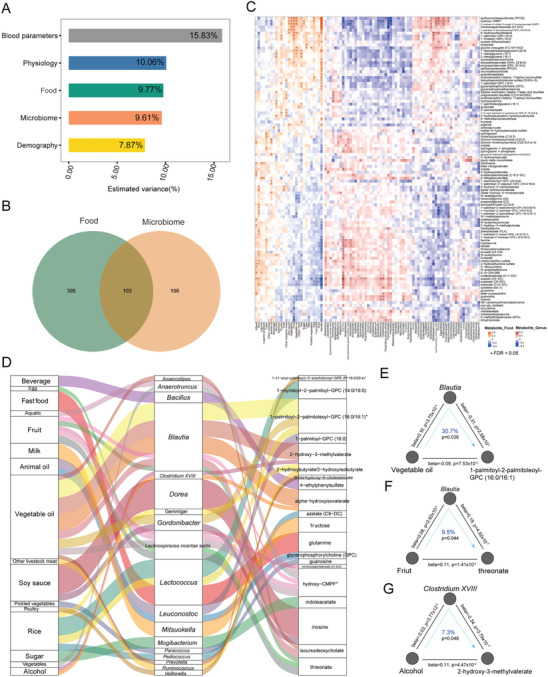
Influence of the microbiome and diet on inter‐individual variation of serum metabolome. A) Contributions of indicated factors to inter‐individual variation in the serum metabolome estimated by the Adonis method (FDR < 0.05). B) Venn diagram indicating the number of metabolites significantly associated with specific foods and gut microbiota genera, as estimated using Spearman's correlation (FDR < 0.05). C) Association of serum metabolites and foods or microbiota genera in HN and GZ province (*n* = 496, FDR < 0.05). D) Mediation links between food, gut microbiota, and serum metabolites showed by parallel coordinates chart that are significant at FDR < 0.05. Shown are foods (left), gut microbiota (middle), and serum metabolites (right). The curved lines connecting the panels indicate the mediation effects. E) Analysis of the effect of vegetable oil intake on the levels of 1‐palmitoyl‐2‐palmitoleoyl‐GPC (16:0/16:1) as mediated by *Blautia*. F) Analysis of the effect of fruit intake on the levels of threonate as mediated by *Blautia*. G) Analysis of the effect of wine intake on the levels of 2‐hydroxy‐3‐methylvalerate through *Clostridium* XVIII. In (E–G), the gray lines indicate the associations. The blue arrowed lines indicate the food effects on serum metabolites mediated by specific genera. The beta coefficient and p values are labeled at each edge. The proportions of indirect effect (mediation effect) and mediation p values are labeled at the center of the ring charts.

To elucidate the effects of diet and microbiota on serum metabolites, the serum metabolome of 496 residents in HN and GZ provinces was tested (Table S6, Supporting Information), and an abundance of 824 serum metabolites was obtained (Figure 1A). The proportions of variance explained by food, physiology, blood parameters, and the gut microbiota across the entire serum metabolome profile were separately calculated. It was shown that all the included variables collectively explained 53.14% of metabolome variations. Among them, food and gut microbiota accounted for 9.77% and 9.61%, respectively (FDR < 0.05, **Figure** **4**A). Next, the associations of each food variable and the gut microbiota with serum metabolites were analyzed using Spearman's correlation, and 408 and 203 metabolites were observed to have a significant association with specific foods and gut bacteria (FDR < 0.05, Tables S7 and S8, Supporting Information). As shown in the Venn diagram, 103 metabolites were simultaneously linked to food and gut bacteria (Figure 4B,). Of these, 351 associations between 103 metabolites and 27 foods, and 389 associations between 103 metabolites and 62 microbiota genera were detected (FDR < 0.05, Figure 4C).

The relationships among food, the gut microbiota, and serum metabolites were characterized, and 33 gut microbes‐mediated links between food and serum metabolites were identified (p_mediation_ < 0.05, Figure 4D; Table S9, Supporting Information). Interestingly, vegetable oil was the food with the most links (eight links) with serum metabolites in these two provinces. Specifically, it led to a decrease in 1‐palmitoyl‐2‐palmitoleoyl‐GPC (16:0/16:1) possibly through increasing the abundance of *Blautia* (30.7%, Figure 4E). *Blautia* mediated the boosting effects of fruit intake on serum threonate levels (9.5%, Figure 4F), a Vitamin C degradation product contributing to improved memory and blood pressure.^[^ ^14^ ^]^ Another interesting example was that drinking wine may increase serum 2‐hydroxy‐3‐methylvalerate by reducing *Clostridium XVIII* (7.3%, Figure 4G).

### 2.5 Serum Metabolites Connect Gut Microbes with Clinical Indexes

**Figure 5:**
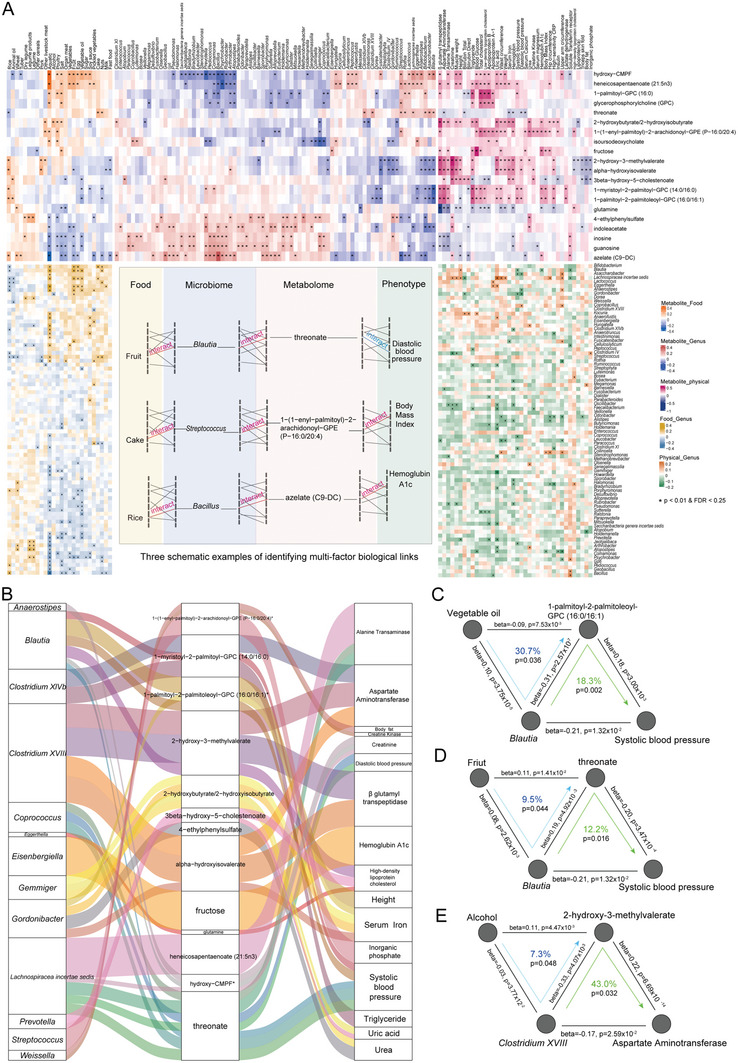
Associations between food, microbiome, metabolites, and host physiological parameters. A) Clustered heatmaps indicating the associations between food, gut microbiome, metabolites, and physiological parameters that are significantly correlated with each other by Spearman's correlation analysis (FDR < 0.25 and *p* < 0.01). Three schematic examples of identifying multi‐factor biological links along the food‐microbiome‐metabolite‐host phenotype axis showing in the box embedded in between the heatmaps. B) Parallel coordinates chart showing the mediation links between gut microbiota, serum metabolites and physiological parameters that were significant at FDR < 0.05. Shown are gut microbiota (left), serum metabolites (middle), and physiological parameters (right). The curved lines connecting the panels indicate the mediation effects. C) Analysis of the causal relationships among vegetable oil intake, *Blautia*, 1‐palmitoyl‐2‐palmitoleoyl‐GPC (16:0/16:1), and systolic blood pressure combined with Figure 4E. D) Analysis of the causal relationships among fruit intake, *Blautia*, threonate and systolic blood pressure combined with Figure 4F. E) Analysis of the causal relationships among wine intake, *Clostridium* XVIII, 2‐hydroxy‐3‐methylvalerate and aspartate aminotransferase combined with Figure 4G. In (C–E), the gray lines indicate the associations. The blue arrowed lines indicate the food effects on serum metabolites mediated by specific genera. The green arrowed lines indicate the specific genera effects on physiological parameters mediated by serum metabolites. The beta coefficient and p values are labeled at each edge. The proportions of indirect effect (mediation effect) and mediation p values are labeled at the center of the ring charts.

Next, the correlation among foods, gut microbes, serum metabolites, and host physiological parameters was explored. Given these data from 496 participants of HN and GZ provinces, pairwise associations between these variables were tested by Spearman's correlation analysis. It was observed that 26 foods, 79 gut bacteria, 20 serum metabolites, and 38 host physiological parameters were significantly correlated with each other (FDR < 0.25 and *p* < 0.01, **Figure** **5**A). Among them, 3946 stepwise interaction linkages were along the food‐microbiome‐metabolite‐host axis (food‐microbiome, microbiome‐metabolite and metabolite‐host phenotypes) (Table S10, Supporting Information). For example, fruit intake was positively correlated with the abundance of *Blautia*, which was positively correlated with serum threonate, a metabolite negatively related to diastolic blood pressure. The intake of cake was also positively associated with the abundance of *Streptococcus*, which was negatively correlated with serum 1‐(1‐enyl‐palmitoyl)−2‐arachidonoyl‐GPE (P‐16:0/20:4) positively related to body mass index. Moreover, rice intake was positively correlated with the abundance of *Bacillus*, which was positively correlated with serum azelate (C9‐DC), while azelate (C9‐DC) was positively related to glycosylated hemoglobin (HbA1c).

Based on the food‐mediated linkage between the gut microbiota and serum metabolites (Figure 4D), the mediating role of serum metabolites between the gut microbiota and physiological parameters was further assessed. Then, 34 mediation links between gut bacteria, serum metabolites, and host physiological parameters were established (p_mediation_ < 0.05, Figure 5B; Table S11, Supporting Information). Interestingly, *Blautia* may reduce systolic blood pressure by reducing the serum level of 1‐palmitoyl‐2‐palmitoleoyl‐GPC (16:0/16:1) (18.3%). However, vegetable oil was a significant food type increasing *Blautia* and also decreasing serum 1‐palmitoyl‐2‐palmitoleoyl‐GPC (16:0/16:1) (Figure 4E), which may be a potential pathway for vegetable oil to suppress systolic blood pressure (Figure 5C). Additionally, fruit also reduced systolic blood pressure by raising the abundance of *Blautia* to improve serum threonate, while threonate mediated the negative effects of *Blautia* on systolic blood pressure (12.2%) (Figure 5D). Combined with Figure 4G, it can be seen that wine may cause an increase in aspartate aminotransferase by increasing the serum level of 2‐hydroxy‐3‐methylvalerate and decreasing the abundance of *Clostridium XVIII* (Figure 5E). Collectively, these results shed light on the ways of how food affects human health.

### 2.6 Aging is Associated with Gut Microbes and Serum Metabolites

**Figure 6:**
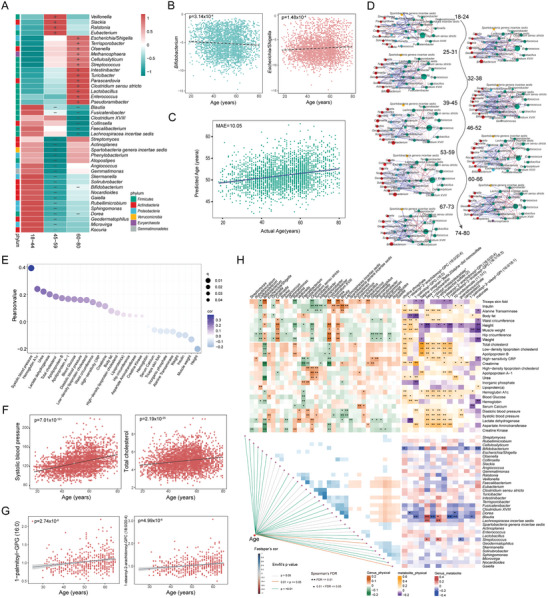
Associations between aging‐related clinical indexes and age‐related gut microbiota, serum metabolites. A) The gut microbiota genera that significantly different in the youth (18–44 years), middle‐aged (45–59 years), and old (60–80 years) age people (FDR < 0.01) using the Envfit method with p values < 0.05. The checkers are colored according to phylum. B) The linear regression of the relationship between relative abundance of *Bifidobacterium* or *Escherichia*/*Shigella* of each individual and age (*n* = 3,224). C) The linear regression actual age and predicted age performed by RF analysis based on microbiota. D) The microbial interaction networks across the entire population at six‐year intervals constructed with FastSpar, FDR < 0.05, cor < mean‐ sd and cor > mean + sd. E) Pearson correlation between age and physiological parameters. The colors of dots indicate the value of cor. The size of dots indicates the value of q. F) The linear regression between the value of systolic blood pressure or low‐density lipoprotein cholesterol of each individual and age. G) The linear regression between the levels of serum 1‐palmitoyl‐GPG (16:0) or 1‐stearoyl‐2‐arachidonoyl‐GPC (18:0/20:4) of each individual and age. H) Spearman's correlation among age‐related gut microbiota, serum metabolites and physiological parameters (FDR < 0.05).

Independent of geography and food variables, age was significantly associated with the gut microbiome as well (Figure 1D; Figure S1B, Supporting Information). The composition of the gut microbiota among youth (aged 18–44), middle‐aged (aged 45–59) and old (aged 60–80) groups was compared, and 34 genera of the gut microbiota were found to be differently distributed among the three groups (FDR < 0.01, **Figure** **6**A). Of them, probiotics *Bifidobacterium, Blautia* and *Lachnospiracea incertae sedis* were enriched in the youth group; *Slack, Eubacterium* and *Ralstonia* were enriched in the middle‐aged group; opportunistic pathogens *Escherichia/Shigella* and *Clostridium sensu stricto* were enriched in the old group. Linear regression analysis suggested that *Bifidobacterium, Blautia*, and *Lachnospiracea incertae sedis* were negatively associated with the increase of age, whereas *Escherichia/Shigella* and *Clostridium sensu stricto* were positively associated with it (Figure 6B; Figure S12A, Supporting Information).

To assess the correlation between gut microbial composition and chronological age, an RF analysis was performed to develop an age prediction model based on microbiota. The model showed desirable performance with a mean absolute error (MAE) of 10.05 years (Figure 6C), which was comparable to recent studies (MAE: 8.1‐10.1/10.6‐13.75).^[^ ^2^, ^15^ ^]^ Subsequently, microbial interaction networks were built across the entire population at an interval of six years, and microbial interactions were demonstrated to increase with age and gradually decrease after the age of 66 (Figure 6D). These results suggest that the gut microbiome may regularly succeed with age.

The correlation of host physiological parameters with age was analyzed by Pearson correlation, and 28 host physiological parameters were shown to be significantly correlated with age in the whole population (Table S12, Supporting Information). Linear regression analysis proved that systolic and diastolic blood pressures, total cholesterol and LDL cholesterol increased significantly with age (p < 0.0001, Figure 6E,F; Figure S12B, Supporting Information). The Pearson correlation between serum metabolites and age in HN and GZ provinces showed a significant correlation between 275 serum metabolites and age (FDR < 0.05, Table S13, Supporting Information). For example, 1‐palmitoyl‐GPG (16:0) and 1‐stearoyl‐2‐arachidonoyl‐GPC were positively correlated with age (p < 0.0001, Figure 6G; Figure S12B, Supporting Information).

Across the population, a Spearman's correlation analysis was performed to test the age‐related connections between the gut microbiota, serum metabolites, and host phenotypes, and 243 correlations were found between 35 age‐related genera and 28 host physiological parameters. In HN and GZ populations, Spearman's correlation analysis demonstrated 22 associations of age‐related serum metabolites with gut microbes and 166 associations with 28 host physiological parameters (Figure 6H). Notably, *Blautia* decreased with age and showed an inverse relationship with several serum metabolites, including 1‐palmitoyl‐GPG (16:0), 1‐ribosyl‐imidazoleacetate and 1‐palmitoyl‐GPE (16:0), which were positively correlated with age. Unlike *Blautia*, these serum metabolites were positively correlated with systolic blood pressure, which increased with age to a large extent (Figure 6H). Therefore, aging‐related hypertension may be induced by the reduction of gut *Blautia* and the increase of these serum metabolites during human aging. In addition, *Bifidobacterium* also decreased with age and showed a negative correlation with multiple age‐related host physiological parameters, namely total cholesterol, creatinine, HbA1c, and age‐related serum metabolites such as campesterol, 5α‐pregnan‐3β and malonate (Figure 6H). This suggests that *Bifidobacterium* is the ideal probiotic to alleviate the age‐related clinical indexes in the elderly.

## 3 Discussion

A majority of microbiome studies to date focused on limited external factors or recruited a small number of participants,^[^ ^3^, ^16^ ^]^ which may risk missing major contributors or ignoring the effects of confounding factors. In this study, 3,224 individuals with information on geography, demography, food, nutrients, physiological parameters, the gut microbiome and serum metabolites were recruited, and the intricate relationships among these factors within this Chinese population were assessed.

The geographic effect has been observed in several cohort‐based studies,^[^ ^3^, ^4^, ^17^ ^]^ but factors mediating the geographic effect on the gut microbiome remain unanswered. It was confirmed that geography explained the largest microbiota variation reported in a Chinese prospective study.^[^ ^18^ ^]^ More importantly, it was also found that the smaller the geographic granularity was, the higher the similarity of the gut microbiota among individuals from 15 provinces would be based on individual geographic locations at different levels, which was similar to a population cohort study in Guangdong Province, China.^[^ ^3a^ ^]^ Unlike the Guangdong population, the effects of area, region, province, city/county, community, latitude, and longitude on the gut microbiota across China were explored. In addition, it was revealed that food variables were strongly associated with geographic information. Mediation analysis showed that diet mediated the effect of geographic locations on the gut microbiota. For instance, *Bifidobacterium* was positively correlated with wheat intake^[^ ^19^ ^]^ and latitude since wheat is the main food in most provinces of northern China. For the first time, it was clearly stated that diet is a driving factor of geographical effect. Of note, the absence of geographic effect was observed in the American Gut Project,^[^ ^20^ ^]^ but such effect was evident in the populations of Congolese^[^ ^3b^ ^]^ and Ecuadorian.^[^ ^17^ ^]^ This observation suggests that living habits and diets tend to converge among people residing in different regions of developed countries. In contrast, more pronounced variations may exist in dietary patterns within different regions of developing countries, like urban and rural areas. As a result, further examination of regional dietary preferences is warranted to more deeply understand the influence of geographic factors on the gut microbiota.

Despite the wide associations of diet and the gut microbiome with individual variations in human plasma or serum metabolome in several cohort‐based studies,^[^ ^2^, ^21^ ^]^ the understanding of the causal relationship among diet, microbiome and metabolism in large‐scale population cohorts remains limited. In the current study, the serum metabolites of individuals from HN and GZ provinces rich in different food sources and Chinese traditional diets were generated to identify 33 gut microbiota‐mediated mediation linkages between food and serum metabolites. Such linkages offer a profound understanding of the interactions between food and microbiome in maintaining human metabolic health, as illustrated by all kinds of glycerylphosphorylcholine that have previously been related to T2D.^[^ ^22^ ^]^ Considering that diet, the gut microbiome, and metabolism are important in human health and disease onset,^[^ ^6^, ^23^ ^]^ 34 mediation links were established between gut bacteria, serum metabolites, and host physiological indexes. By combining the mediation relationships between food, microbiome, and metabolites, several linkages were discovered along the food‐microbiome‐metabolites‐physiological index. Among them, fruit intake can reduce systolic blood pressure by increasing the abundance of *Blautia* and improving the serum level of threonate, a degradation product of Vitamin C conducive to improving memory and blood pressure.^[^ ^14^ ^]^ Unlike the findings of this study, 13 mediation linkages for the impact of diet on metabolites through microbiome were established in the population‐based Lifelines DEEP study.^[^ ^8^ ^]^ Among them, *Ruminococcus* species vSV (300–305 kb) encoding adenosine triphosphatase mediated the impact of fruit consumption on the plasma level of urolithin B, but increased the plasma level of LDL via tyrosol 4‐sulfate, a uremic toxin, when identifying Lifelines‐DEEP samples taken four years apart.^[^ ^12^ ^]^ Due to data characteristics, microbial primary metabolic gene clusters and genomic structural variants cannot be identified. Notwithstanding, these mediating relationships may differ among distinct populations owing to variations in diet, ethnicity, and other factors.

In this cohort, age was also a significant variable strongly associated with microbial variance. It was found that *Bifidobacterium*, *Blautia* and *Lachnospiracea incertae sedis* were negatively associated with the increase of age, while opportunistic pathogens *Escherichia/Shigella* and *Clostridium sensu stricto* were positively associated with it. Among age‐associated metabolites, 1‐palmitoyl‐GPG (16:0) and 1‐stearoyl‐2‐arachidonoyl‐GPC were positively correlated with age, which had not been reported before. In addition, age‐related clinical indicators were demonstrated to have an association with age‐related changes in the gut microbiota and serum metabolites.^[^ ^24^ ^]^ For example, decreased gut *Blautia* and increased serum 1‐palmitoyl‐GPG (16:0) may be bound up with hypertension. These results provide new insights into the characteristics of aging and age‐related diseases.

Several limitations exist in this study. It is recommended to include appropriate positive and negative sequencing controls to avoid the possibility of misinterpreting the composition of the microbial community. In consideration of the compositional nature of microbiome data, microbiome research should widely adopt satisfactory approaches such as Aitchison, PCA and SparCC rather than classical analysis methods, including Bray‐Curtis dissimilarity, principal coordinate analysis and linear regression designed to handle counting data in ecology. In addition, mediation analysis about the impact of diet and gut microbiota on serum metabolites in larger populations may further strengthen the observations of this study and better determine underlying biological significance. At last, this study is based on correlative analysis, and future experimental studies are necessitated to unravel the accurate relationships between the given food, the gut microbiota, serum metabolites, physiological indexes, and the mechanistic aspect.

## 4 Conclusion

First, a panel of gut microbiota associated with geography, food, and age in a large cohort across the wide geographic scale in China was identified in the present study. Second, it was demonstrated that food is the main mediating factor of geographic locations on the gut microbiota and modulates human serum metabolites. Third, the impact of geography, diet, and gut microbiota on serum metabolites and host health in a Chinese population was validated, and a comprehensive resource that can guide follow‐up studies aimed at designing preventive and therapeutic strategies for human health was provided.

## 5 Experimental Section

### Ethical Permission and Sample Collection

The present study is based on the China Health and Nutrition Survey (CHNS), a prospective population‐based survey that covers geography, food, nutrients, and health phenotypes.^[^ ^25^ ^]^ The CHNS protocol gained the approval of the Institutional Review Boards of the Chinese Center for Disease Control and Prevention (No. 201 524) and the University of North Carolina at Chapel Hill (No. 07–1963). All participants signed an informed consent form before sample collection.

Fasting blood samples were gathered, stored in dry ice and sent to the laboratory for storage at −80 °C. After being centrifuged within 48 h, the plasma was stored at −80 °C for later use. Fecal samples were collected following standard procedures,^[^ ^26^ ^]^ kept in a −20 °C freezer within 20 min and stored in a −80 °C laboratory.

### Metadata Collection

Metadata mainly includes demographics, geography, food, nutrients, and physiological parameters (Tables S1–S3, Supporting Information). Each individual had lived in the sampling place for generations. In this study, the information on community, city/county, province, region, area, longitude, and latitude was translated according to the detailed residential location of individuals. The weighing method was adopted to record the consumption of household oil and condiments, and the query method was employed to obtain food consumption for three consecutive days. Data on nutrient intake were calculated based on food consumption data and the Chinese Food Composition Table.^[^ ^27^ ^]^ Height, weight, waist and hip circumferences, blood pressure, and other indicators were measured by use of uniformly distributed instruments. The collected fasting plasma samples were sent to a third‐party testing institution for testing to obtain blood glucose, total cholesterol, LDL cholesterol, and other blood parameters.

### Gut Microbiome Sequencing and Preprocessing

The classical V4 region of 16S ribosomal ribonucleic acid of the gut microbiome was sequenced on a Novogen's Illumina HiSeq PE‐250 platform according to standard operation procedure. Uparse pipeline in Usearch11 was applied to obtain the operational taxonomic unit (OTU) table. The taxonomic assignment of OTUs was predicted by the Naive Bayesian Classifier algorithm based on the Ribosomal Database Project (RDP) database. Sva packages (version 3.40.0) were used for reducing batch effects for high‐throughput data. Besides, α‐(Chao1, Shannon and observed OTUs, and Faith's phylogenetic diversity) and β‐diversity indices (Bray_curtis) were computed based on the flattened OTU table.

### Variance Analysis

Adonis, ANOSIM, MRPP, and dbRDA were applied to the R‐based vegan package (version 2.6‐2). Each factor was counted individually. P‐values were determined from 1,000 permutations and adjusted to obtain FDR values. Significant results were filtered based on FDR < 0.05. Adonis R^2^ values were utilized for estimating the variance of gut microbiome contributions from city/county and community groups across provinces.

### Similarity Analysis

The average Bray_Curtis distance and Pearson value of samples within and without the group were counted under five geographic factors. The southernmost or northernmost sample was used as a reference to calculate the distance. Linear regression was performed to evaluate the association between the actual distance and similarity of samples.

### LDA and PCA

LDA (MASS package, version 7.3‐55)^[^ ^28^ ^]^ was applied to reduce the dimensions of data, and PCA^[^ ^28^ ^]^ was used to perform unsupervised clustering on the reduced dimension data by province and region groups.

### Characteristics of Microbiome in Different Geographic Granulates

MaAsLin was implemented in the MaAsLin2 package (version 1.6.0)^[^ ^29^ ^]^ to determine the multivariate correlations between metadata and microbial traits. The union of specific genera in 15 provinces was counted, and the network of each province was constructed with FastSpar software (version 1.0.0) in Python.^[^ ^30^ ^]^ Statistically significant results (FDR < 0.05, cor < mean – sd and cor > mean + sd) for each network were visualized in Cytoscape software (version 3.10.1). RF analysis (RF package, version 4.7‐1.1)^[^ ^31^ ^]^ was performed by specific genera, and the leave‐one‐out method was employed to predict which province an individual came from.

### Analysis of Geographic Ranges, Food Factors, and the Gut Microbiome

Variables filtered by the Adonis method and different geographic ranges were analyzed by Cramer's V,^[^ ^32^ ^]^ and significant results (p < 0.05 and Cramer's V > 0.2) were plotted using the ggcor package (version 0.9.8.1). The confirmed results of Boruta analysis (Boruta package, version 7.0.0) between genera and food were shown with the pheatmap package (version 1.0.12). Mediation between latitude information, food and genera was implemented in the mediation package (version 4.5.0).^[^ ^33^ ^]^ The effects of longitude, age, and gender were corrected, and p values < 0.05 were considered to show statistical significance.

### Serum Metabolome Analysis

An analysis was made of the serum metabolome by the Metabolon platform using ultrahigh performance liquid chromatography‐tandem mass spectrometry (UPLC‐MS/MS), followed by the processing of samples using an automated MicroLab STAR system (Hamilton Company). The hardware and software of Metabolon were used to perform peak integration, data extraction, and quality control. Chemicals were identified by comparing purified standards (retention time/index, mass‐to‐charge ratio, and chromatographic data) with unknown entities in the Metabolon library.

### Correlation Analysis Among Multiple Parameters

Metabolite metadata were analyzed by the Adonis method for variance with FDR < 0.05. Relationships between food, the gut microbiome, metabolites, and physiological parameters were parsed using Spearman's correlation with FDR < 0.25 and p value < 0.01. All correlation results were presented as an integrated heatmap using the ComplexHeatmap package (version 2.8.0). Mediating effects among food, the gut microbiota, metabolites, and physiological parameters were analyzed, separately.

### Analysis of Age‐Related Variables

The MaAslin method was adopted to analyze the differential gut microbiota of the three age groups. RF analysis was conducted to predict the age of each individual, and the regression results between the actual and predicted ages were shown. The network was constructed with FastSpar, *p* < 0.01, cor < mean – sd and cor > mean + sd. Pearson correlation was used for analyzing the association of age with physiological parameters or metabolites. Associations between age groups and different genera were analyzed using the Envfit method with p values < 0.05.

For detailed methods see the Supporting Information.

## Conflict of Interest

The authors declare no conflict of interest.

## Author Contributions

J.G.Z., H.B.Q., M.H.L., and Z.H.W. contributed equally to this work. Q.F., B.Z., and H.J.W. designed the study; M.H.L. and H.B.Q. performed the data analysis; H.B.Q. and Q.F. drafted the manuscript; Q.F., H.B.Q., J.G.Z., H.J.W., and M.H.L. revised the manuscript; J.G.Z., Z.H.W., and X.F.J. collected the data; S.F.D., C.S., W.W.D., Y.F.Oy., F.F.H., H.R.J., L.L., J.B., Y.L.W., and X.F.Z. participated in acquisition of data. T.Y.S., M.F.Z., and P.P.W. participated in interpretation of data; All authors participated in the critical revision of the manuscript and approved the final version of the manuscript.

## Supporting information

Supporting Information

Supplemental Tables

## Data Availability

The data that support the findings of this study are openly available in the Genome Sequence Archive in National Genomics Data Center at https://www.bigd.big.ac.cn/gsa, reference number 13939.
