# Supplementary material for: Diet Mediate the Impact of Host Habitat on Gut Microbiome and Influence Clinical Indexes by Modulating Gut Microbes and Serum Metabolites
Source: Adv Sci (Weinh). 2024 Mar 13;11(19):2310068. doi: 10.1002/advs.202310068 (PMC11109649; doi:10.1002/advs.202310068)
Supplement: Supplementary file 1 — Supporting Information [file ADVS-11-2310068-s001.pdf]

ADVANCED SCIENCE
Open Access

Supporting Information

for *Adv. Sci.*, DOI 10.1002/advs.202310068

# Diet Mediate the Impact of Host Habitat on Gut Microbiome and Influence Clinical Indexes by Modulating Gut Microbes and Serum Metabolites

*Jiguo Zhang, Houbao Qi, Meihui Li, Zhihong Wang, Xiaofang Jia, Tianyong Sun, Shufa Du, Chang Su, Mengfan Zhi, Wenwen Du, Yifei Ouyang, Pingping Wang, Feifei Huang, Hongru Jiang, Li Li, Jing Bai, Yanli Wei, Xiaofan Zhang, Huijun Wang*, Bing Zhang* and Qiang Feng**

## Detailed Methods

**Ethical permission and sample collection.** This study is based on China Health and Nutrition Survey (CHNS), a prospective population-based survey that covered geography, food, nutrient, and health phenotypes. The CHNS protocol was approved by the ethnical committee of Institutional Review Boards of the Chinese Center for Disease Control and Prevention (number 201524) and the University of North Carolina at Chapel Hill and the National Institute for Nutrition and Health (number 07-1963). All participants signed the informed consent form before sample collection.

On the first day of the dietary survey, the investigator sent the consumables package to the respondents of each household and explained the purpose and use time of each consumable to the respondents. Each respondent collected stool samples at any time during the second day. Due to the lack of samples caused by forgetting, not defecating or going out, stool samples were gathered at any time on the third day.

The respondents wore disposable gloves, picked up the middle stool without pus, blood, water and urine with a plastic spoon, put it into a stool box until it was full (0.5 cm from the box edge), and closed the lid tightly. The collection of the second stool box was also completed as described above. The respondents sent stool boxes to the village hospital or community health station within 5 minutes. Under the guidance of the person in charge of the village hospital or community health station, stool boxes were stored in a refrigerator (with ice or a temperature of -20 °C). The respondents made a registration on the biological sample collection roster, with a focus on recording the defecation, delivery and freezing time (hour: minute) of stool samples. All stool samples were uniformly transferred by a third-party testing company to a -80 °C refrigerator in the laboratory for storage.

**Serum metabolome generation.** In this step, 10ml fasting blood samples were collected from subjects aged over seven, including one 4 ml ethylene diamine tetraacetic acid (EDTA) blood sample and two 4ml separated gel coagulant blood samples. Blood samples were stored in the refrigerator (-2 - 8 °C) with a ratio of 1:1 for the sample to dry ice, and sent to the local laboratory for storage within 3 hours. The plasma was centrifuged within 48 hours and stored at the temperature of - 80 °C until treatment. All stations observed the same scheme of blood sample collection, storage and processing.

Non-targeted metabolomics analysis was conducted using the Metabolon platform (Durham, North Carolina, USA) consisting of an integrated UPLC-MS/MS (Durham, North Carolina, USA) at the partner campus of Metabolon in China. An automated MicroLab STAR system (Hamilton Company) was utilized to process samples, with several recovery standards and controls as technical replicates for quality control. Methanol was used for removing protein, dissociating small molecules and recovering chemically diverse metabolites. A Waters Acquity UPLC (Waters, Milford, MA) and a Thermo Scientific Q-Exactive high-resolution MS (Thermo Fisher) with heated electrospray ionization (HESI-II) and Orbitrap mass analyzer (with a mass resolution of 35,000) were used in all methods. Peak integration, data extraction, and quality control were performed using the hardware and software of Metabolon built under Microsoft. NET framework. Chemicals were identified by comparing purified standards (retention time/index, mass-to-charge ratio and chromatographic data) with unknown entities in the Metabolon library.

**Metadata collection.** A total of 109 factors were contained, including geography, demography, food, nutrients and physiology and blood parameters (Table S1). Each individual has lived in the sampling place for generations, and the information on community, city, province, region, area, longitude and

latitude was translated based on the detailed residential location of individual.

In this survey, the weighing method was used to record the consumption of household oil and condiments, and the inquiry method was adopted to acquire the food consumption of the respondents for three consecutive days. The investigator entered the house every day to record the purchase, disposal and personal consumption of household food and collected the data from the dietary survey on the day of the investigation. Family members assisted in recording food consumption, and the investigator learned about food consumption in combination with the auxiliary record.

Physical measurement refers to measuring height, weight and body composition, waist, hip and upper arm circumferences, triceps skinfold thickness, blood pressure, body temperature and other indicators with uniform distributed instruments following the uniform method. It was arranged on the morning of the first day after the household survey. Respondents of the same community were organized by the survey team to conduct unified physical measurement in the community health center, neighborhood committee or other appropriate places.

**Gut microbiome sequencing and preprocessing**. The classical V4 region of 16S rRNA of the gut microbiome was sequenced on an Illumina HiSeq PE-250 platform under standard operation procedure in Novogen company. Uparse pipeline in Usearch11 was utilized to obtain the OTU table. The low-quality reads of the raw sequencing data were discarded with a parameter fastq_maxee of 1.5 to get highly accurate OTU sequences. The filtered reads were clustered into OTUs at a cutoff of 97% using the Uparse algorithm implemented in Usearch11. Then, an OTU table was created by the otutab command. The taxonomic assignment of bacterial OTUs was predicted using the Naive Bayesian Classifier algorithm based on the RDP v16 database. Finally, the OTU table and taxonomy information were merged for downstream biostatistical analysis.

Due to the differences in sampling time, location and operators, the data obtained may have batch effect. Hence, sva packages (version 3.46.0) were used to estimate the source of batch effects and reduce the batch effects of high-throughput data. The adjusted p-value of the 6,871 OTUs obtained from the original sequencing data were calculated, and the adjusted p-values less than 0.05 were retained. Finally, 1,129 OTUs were removed and 5,742 were retained for subsequent analysis. In addition, α- and β- diversity analyses were conducted using the OTU count table that was previously down sampling according to the minimum number of reads in order to reduce errors in the evaluation of indicators such as microbial diversity. We used the qiime diversity core-metrics-phylogenetic command in qiime2(2020.2) to calculate the α-diversity indices including Observed_OTUs, Shannon and faith_PD. Then the Chao1 and Simpson indices were calculated through the parameter p-metric of the qiime diversity alpha command. β-diversity which concludes bray_curtis, jaccard distance were used to measurement the difference degree of microbial abundance distribution among samples. The subsequent data analyses were performed in R v4.3.1.

**Variance analysis.** Continuous data were grouped in quartiles, and the date of blood parameters were divided into low, normal and high groups. At the OTU level, Adonis, ANOSIM, MRPP and dbRDA were applied with the vegan package (version 2.6-2), based on Bray-Curtis distance to evaluate the relationship between variables and the gut microbiome. Each factor was counted separately, and p values were determined according to 1000 permutations. All p values were adjusted to obtain FDR values. The results of Adonis $R^2$ were selected to make statistics for the five factor groups. Finally, the significant results were filtered as per FDR < 0.05 for the four methods. The $R^2$ value of the Adonis method was measured to estimate variance of the gut microbiome that was contributed by city and community groups in each province.

**Similarity analysis**. The average Bray-Curtis distance and Pearson value of samples within and without the group were counted to compare the sample similarity under five geographical factors. The southernmost or northernmost sample was used as a reference to calculate the distance with other samples. Linear regression was performed to evaluate the association between the actual distance and similarity of samples. The p value $< 0.05$ was considered as a significant result.

**LDA and PCA analysis**. Linear Discriminant Analysis (LDA) (MASS package, version 7.3-55) was applied to reduce the dimensions of the data, and Principal Component Analysis (PCA) analysis was used for unsupervised clustering of the reduced dimension data at the province and region groups. The clustering results of samples were displayed on the PC1 axis.

**Characteristics of microbiome in different geographical granulates**. At the genus level, the gut microbiome composition and characteristics of different geographic granulates were compared through diversity and cluster analysis in province, region and area. MaAsLin was implemented in the MaAsLin2 package (version 1.6.0) to determine the multivariate correlation between metadata and microbial characteristics. Different genera between one province and the other 14 provinces were calculated among 15 provinces, respectively. In the MaAsLin model, the input data was relative abundance genera data, the transform parameter was "AST", and the Age, Gender, Smoker, Pa are selected the correction factors. In each province, the genera differing from more than two-thirds of the provinces were defined as specific genera. Furthermore, the union of specific genera in 15 provinces were counted, and the network of each province was constructed with Fastspar software (version 1.0.0) in Python. The statistically significant results (FDR $< 0.05$, cor $<$ mean - sd and cor $>$ mean + sd) of each network were visualized in Cytoscape software (version 3.10.1). RF analysis (randomForest package, version 4.7-1.1) was carried out by the union of

specific genera, and the 10-fold cross validation method was used to predict which province the sample belonged to.

**Analysis of geographical ranges, food factors and the gut microbiome**. Variables filtered by the Adonis method and different geographical ranges were analyzed by Cramer's V analysis, and the significant result ($p < 0.05$ and Cramer's $V > 0.2$) were plotted with the corrplot package (version 0.920). The confirmed results of Boruta analysis (Boruta package, version 7.0.0) between genera and food were shown with the pheatmap package (version 1.0.12). At the community level, the longitude and latitude information of the respondents was collected, and the mediation effects of latitude information, food and genera were analyzed, with food factors as the intermediary variable in the mediation package (version 4.5.0). The influence of longitude, age and gender was corrected, and p values $< 0.05$ were considered to show statistical significance.

**Correlation analysis among multiple parameters**. The variance of metadata for metabolites was analyzed by the Adonis method, and $FDR < 0.05$ was considered to show statistical significance. The relationship among food, the gut microbiome, metabolites and physiology parameters was analyzed by Spearman correlation analysis with $p < 0.01$ and $FDR < 0.25$. All the correlation results were presented in the form of an integrated heatmap using the ComplexHeatmap package (version 2.8.0). Mediation analysis was performed on food, genera of the gut microbiome, physiological indicators and metabolites filtered previously. The Age, Gender and Province are selected as the correction factors and the results of the mediating effect ($p < 0.05$) were retained. In addition, the linear regression relationship of the three was calculated.

**Analysis of variables related to age**. The respondents were classified into three age groups: young (aged 18-44), middle-aged (aged 45-59), and old people (aged 60-80), and the differential genera of

the gut microbiota among three age groups were analyzed by the MaAslin method, with young people as the control. RF analysis was carried out according to these differential genera and leave-one-out cross validation was used to predict the age of each individual. The regression results between the actual and predicted age were displayed. The respondents at the age of 18-80 were divided into nine age groups at an interval of six years. Next, the network of nine age groups based on differential genera was constructed with Fastspar, and filtered according to $p < 0.01$, cor < mean-sd and cor > mean +sd. Pearson correlation was used for analyzing the relationship between age and physiological parameters in the whole cohort and that between age and metabolites in Hunan (HN) and Guizhou (GZ) provinces. In the whole population, the envfit method (vegan package, version 2.6-2) was employed to analyze the association between age groups and different genera with p value < 0.05,. Spearman correlation was utilized for investigating the relationship between genera, metabolites and physiological parameters. All the correlation results were presented in the form of an integrated heatmap using the ComplexHeatmap package (version 2.8.0).

**Supplemental Figures**

**Figure S1**

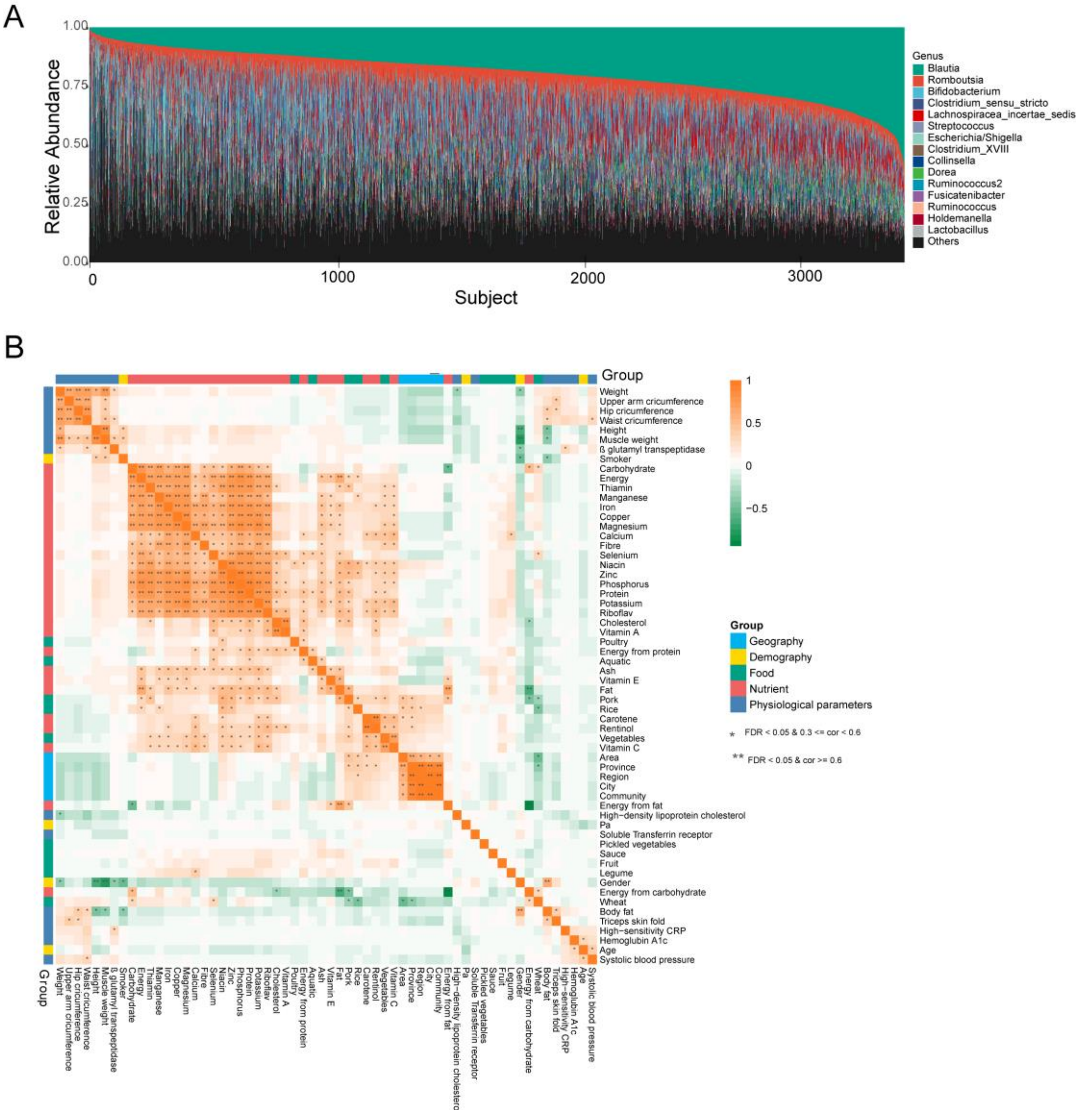


**Figure S1 Top 15 genera composition and variables relationships.** (A) The relative abundances of the top 15 genera of the cohort ($n$ = 3,224). (B) The spearman correlation between significant indicators of all samples ($n$ = 3,224) in adonis results (FDR < 0.05). The statistically significant

results (FDR < 0.05, cor >= 0.3) of spearman are visualized in heatmap.

**Figure S2**

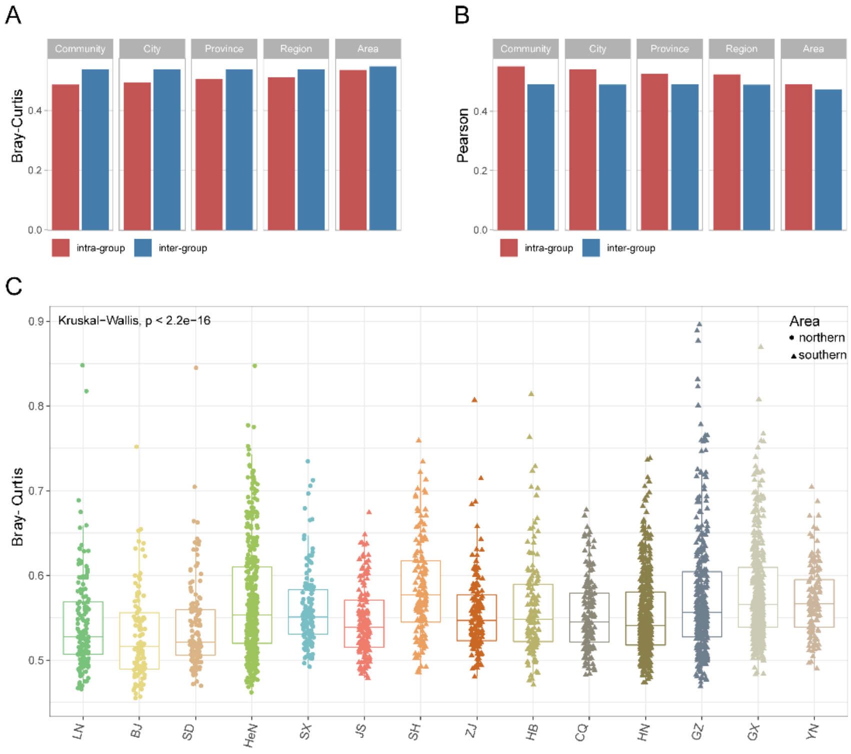


**Figure S2 Characteristics of microbiome in different geographical granulates.** (A-B) The average Bray-Curtis distance and Pearson value of samples (*n* = 3,224) within or without the group under the five geographical ranges. (C) Taking Heilongjiang Province (*n* = 235) as a reference, the Bray-Curtis distance of samples between other provinces and Heilongjiang Province. Each point in the Bray-Curtis dissimilarity matrix represents the average dissimilarity between the microbial communities of one sample from another location and each sample from Heilongjiang Province. The provinces are arranged from north to south, and the shape of the point represents that the province is north or south. Kruskal-Wallis test is carried out among groups and the significant results are shown ($p < 0.05$).

**Figure S3**

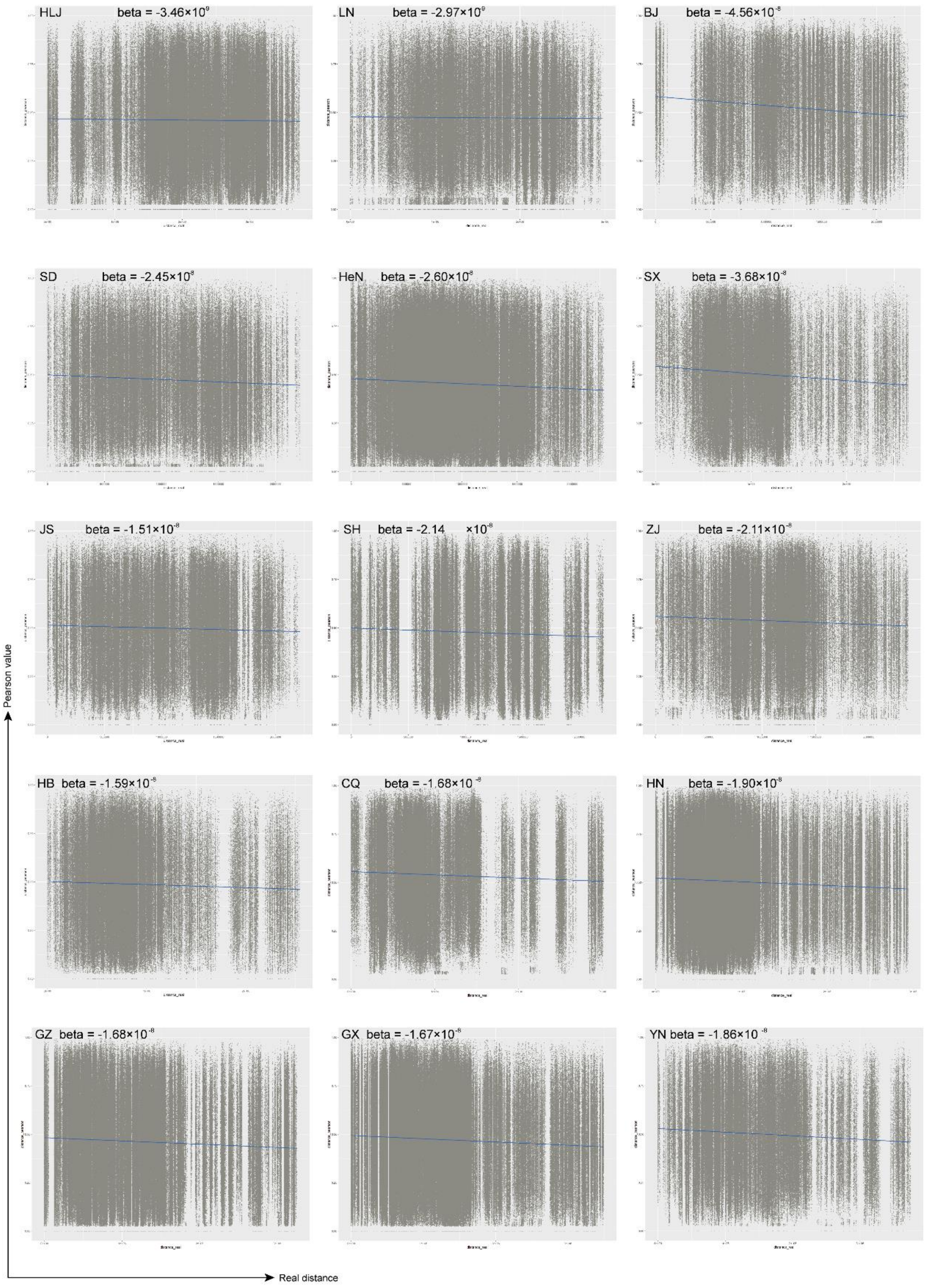
HLJ beta = -3.46×$10^{9}$
LN beta = -2.97×$10^{-9}$
BJ beta = -4.56×$10^{-8}$
SD beta = -2.45×$10^{-8}$
HeN beta = -2.60×$10^{-8}$
SX beta = -3.68×$10^{-8}$
JS beta = -1.51×$10^{-8}$
SH beta = -2.14 ×$10^{-8}$
ZJ beta = -2.11×$10^{-8}$
HB beta = -1.59×$10^{-8}$
CQ beta = -1.68×$10^{-8}$
HN beta = -1.90×$10^{-8}$
GZ beta = -1.68×$10^{-8}$
GX beta = -1.67×$10^{-8}$
YN beta = -1.86×$10^{-8}$
Pearson value
Real distance

**Figure S3 The linear regression of actual distance and Pearson value between other samples and the control sample.** Each province is as a reference, and the Pearson value between samples from other provinces (n = 14) and samples of the control province is calculated. The x and y axes indicate real distance and Pearson value between samples, respectively.

**Figure S4**

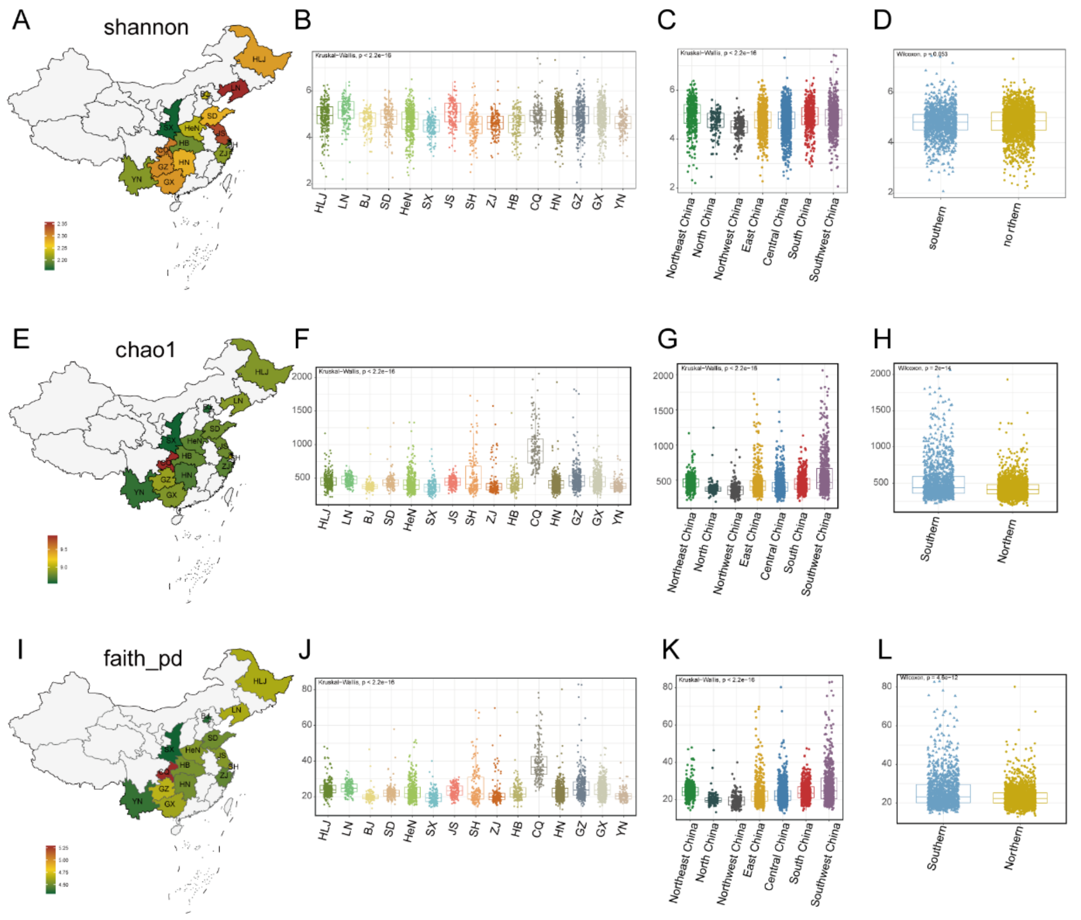


**Figure S4 α diversity in different geographical ranges.** (A) Mean value of shannon diversity index of 15 provinces. (B-D) Shannon diversity index in province, region and area group. Multi-group comparison is performed with Kruskal-Wallis test ($p < 0.05$), and the two groups are performed with Wilcoxon test ($p < 0.05$). (E-I) Chao1 and faith_pd alpha diversity index in province, region and area group of all samples ($n = 3,224$).

## Figure S5

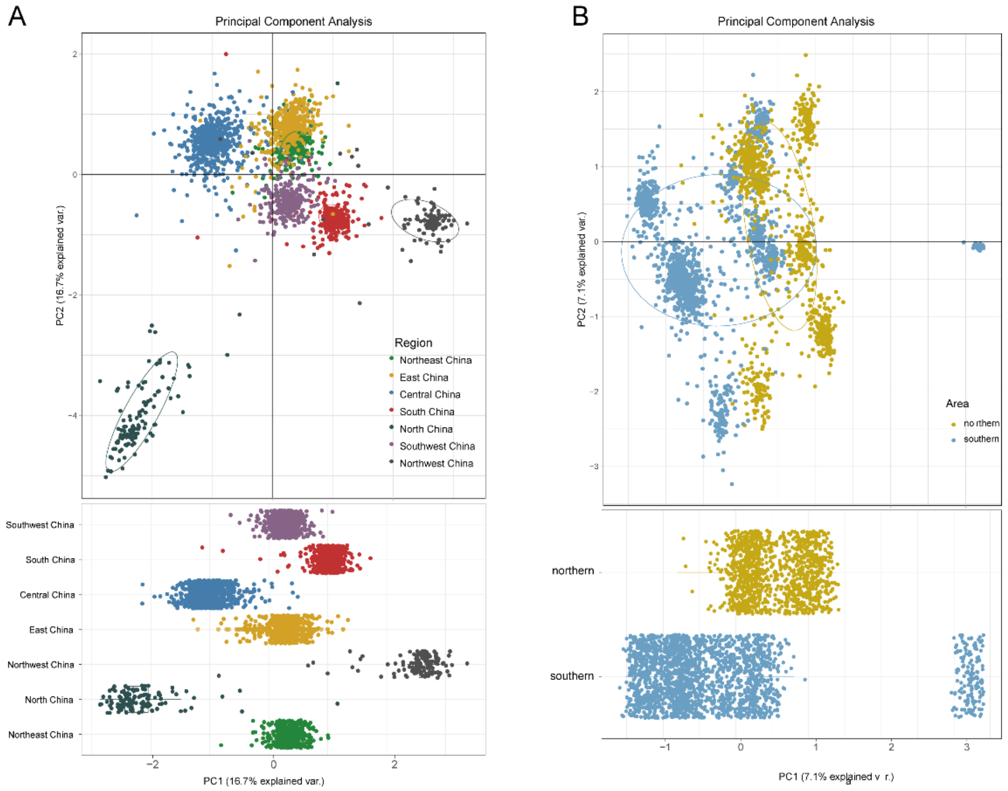


**Figure S5 Sample clustering based on Linear Discriminant Analysis (LDA) and principal component analysis (PCA).** (A) PCA map showing the OTU-based sample clustering in region group, and the samples (*n* = 3,224) distribution in different regions are showed on PC1 axis. (B) PCA clustering based on provinces and coloring with northern and southern group.

## Figure S6

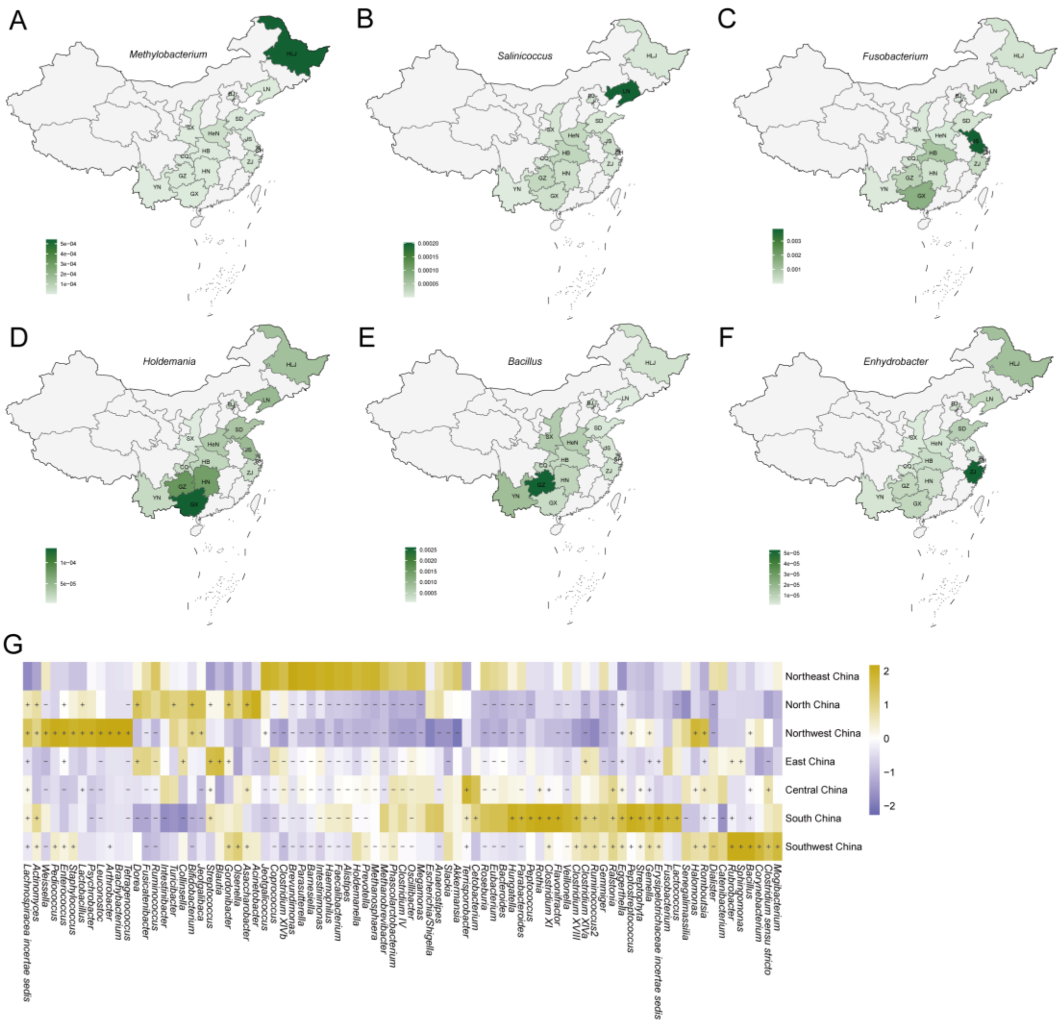


**Figure S6 Characteristic bacteria in different geographic ranges.** (A-F) The mean relative abundance of specific genera in 15 provinces are showed in map. The color depth indicates the abundance of bacteria. (G) The union different genera in region group based on MaAslin analysis (FDR < 0.01). Heatmap shows the relative abundance of genera in different regions.

**Figure S7**

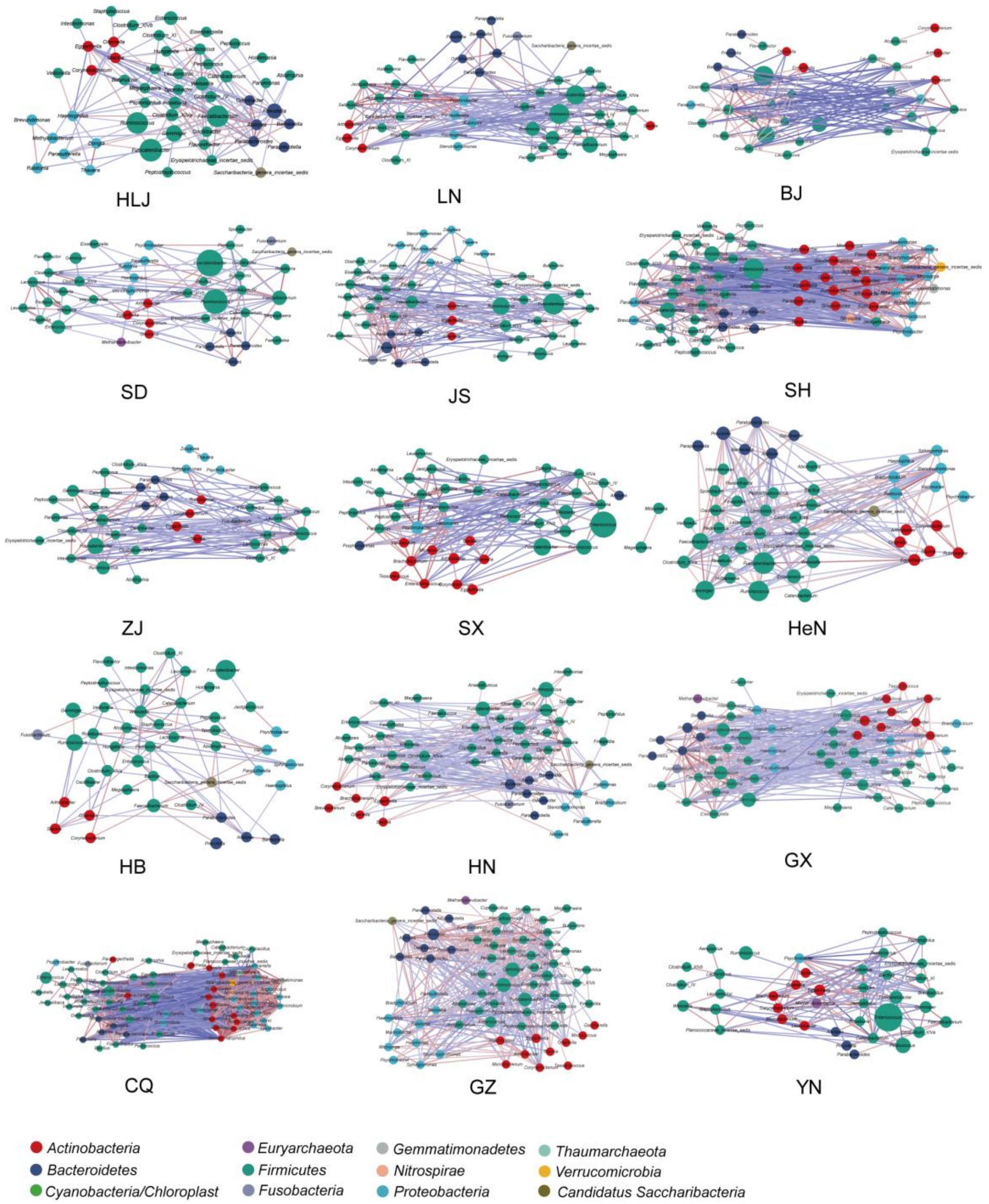


**Figure S7 The microbial interaction network of each province.** The union of specific genera in 15 provinces are counted, and the network of each province is constructed with fastspar. The size of point represents the relative abundance of genera in the province and the points are colored using phylum that the genus belongs. The statistically significant results (FDR < 0.05, cor < mean - sd and cor > mean + sd) of each network are visualized in the network. The red line indicates

positive correlation, and blue line indicates negative correlation. The number of samples in each province: HLJ (*n* = 235), LN (n = 141), BJ (*n* = 115), SD (*n* = 131), JS (*n* = 146), SH (*n* = 140), ZJ (*n* = 142), SX (*n* = 140), HeN (*n* = 390), HB (*n* = 134), HN (*n* = 457), GX (*n* = 433), CQ (*n* = 148), GZ (*n* = 342), YN (*n* = 130).

**Figure S8**

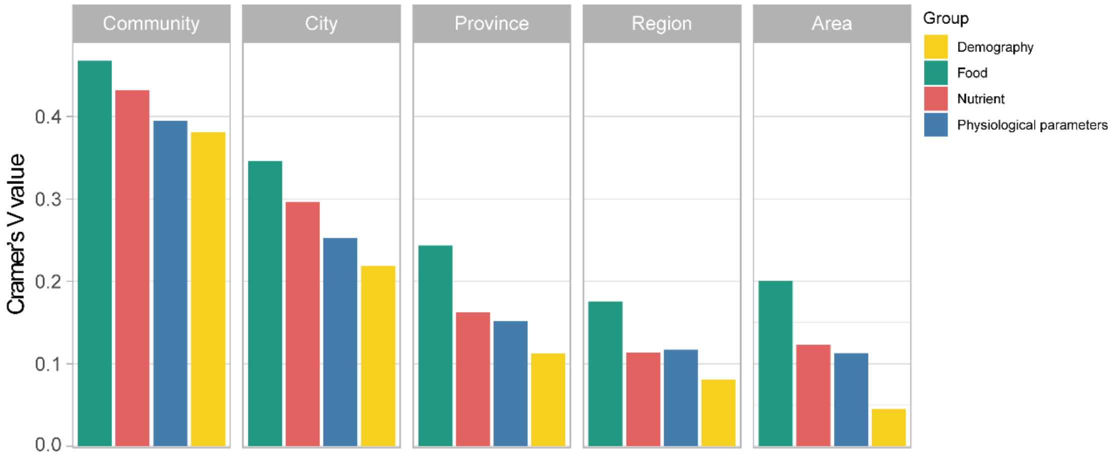


**Figure S8 Relationship between variables and different geographic ranges.** The bar plot shows the Cramer's V values between variables and five geographic ranges. The height of the bar plot represents the average value of the relationship between the variables group and the geographic ranges.

**Figure S9**

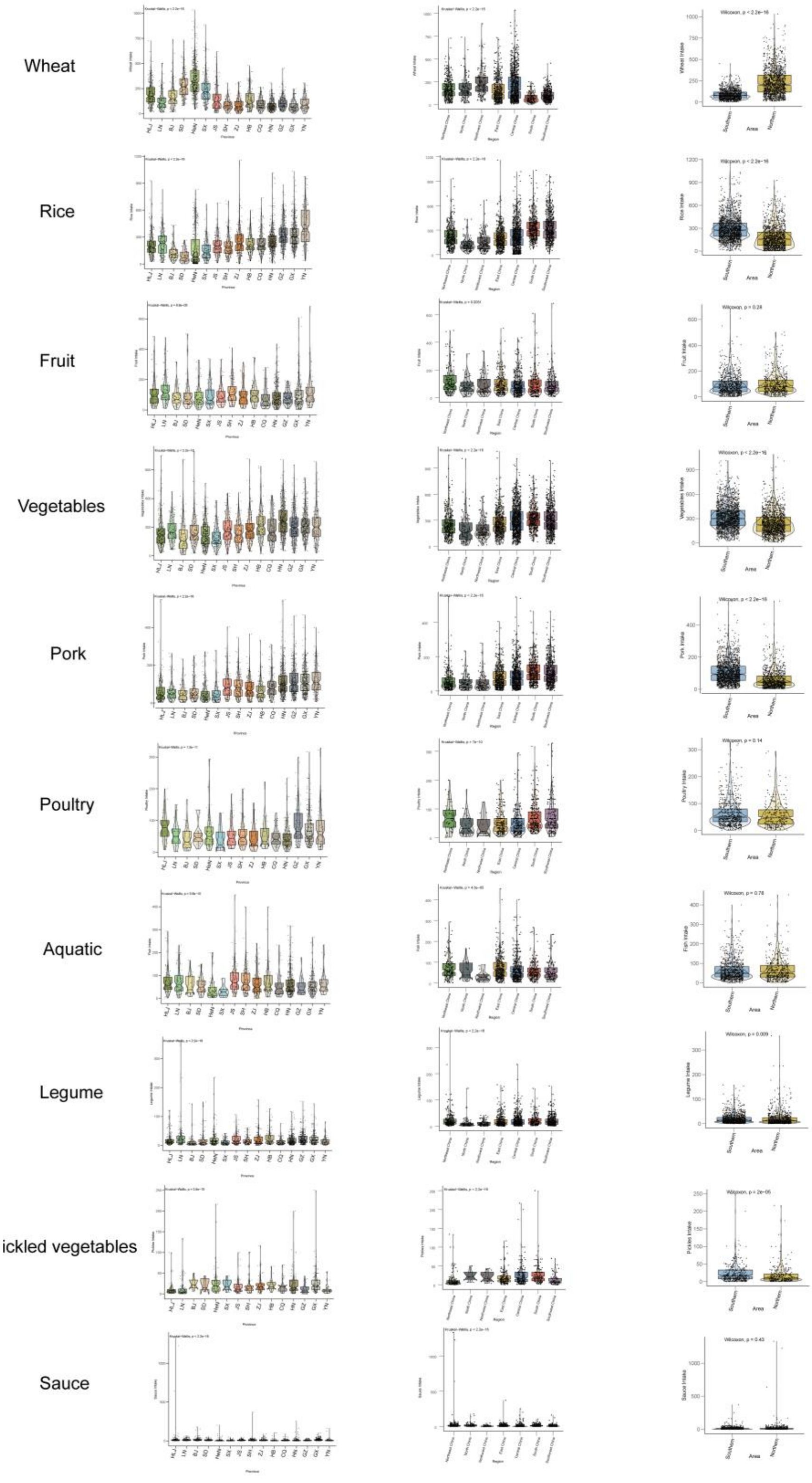


**Figure S9 Foods intake in different geographic ranges.** The intake of 10 foods significantly related (Cramer's V, $p < 0.05$) to geographic ranges in province, region, area group. Multi-group comparison is performed with Kruskal-Wallis test ($p < 0.05$), and the two groups are performed with

wilcoxon test ($p < 0.05$).

**Figure S10**

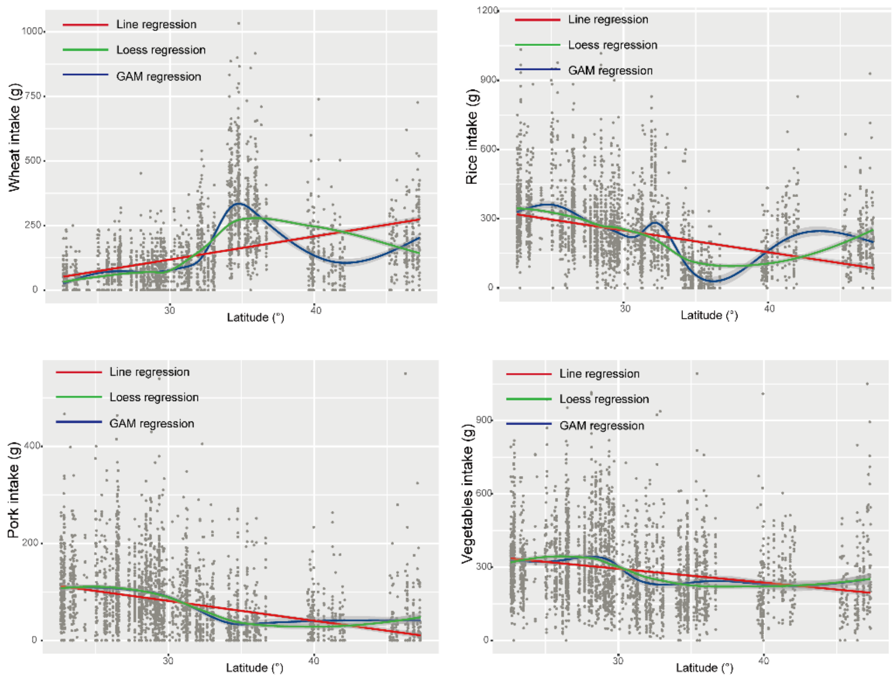


**Figure S10 Relationship between latitude and foods.** Regression results of main foods and latitude. The x and y axes indicate latitude of samples location and food intake of samples (*n* = 3,224), respectively. The red, green, blue line represent line regression, loess regression, and the Generalized Additive Model (GAM) regression, respectively.

**Figure S11**

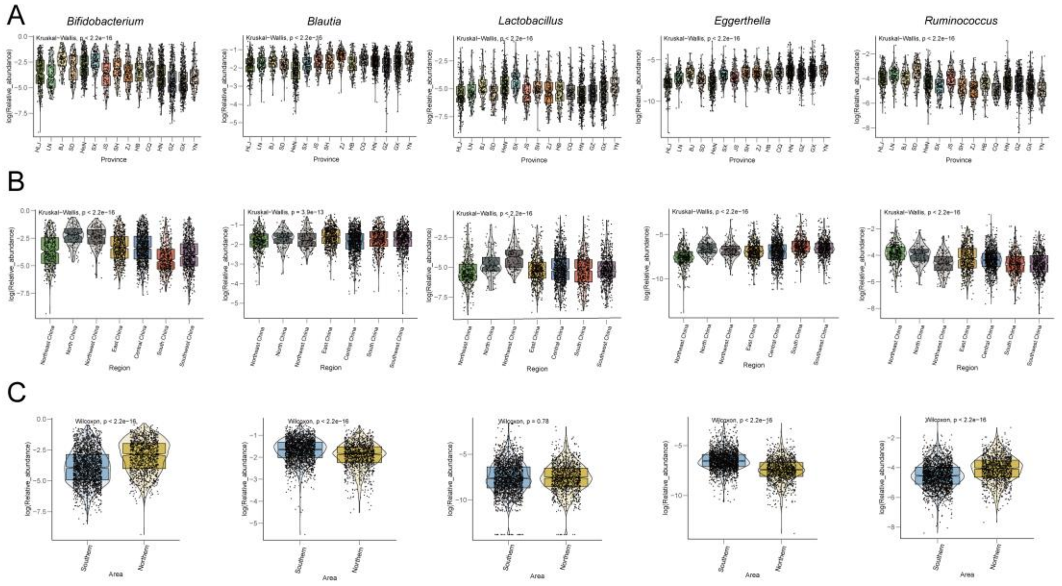


**Figure S11 Genera distribution in different geographic ranges.** Distribution of genera closely related to food (Boruta analysis) of all samples ($n$ = 3,224) in province, region, area group. Multi-group comparison is performed with Kruskal-Wallis test ($p < 0.05$), and the two groups are performed with Wilcoxon test ($p < 0.05$).

**Figure S12**

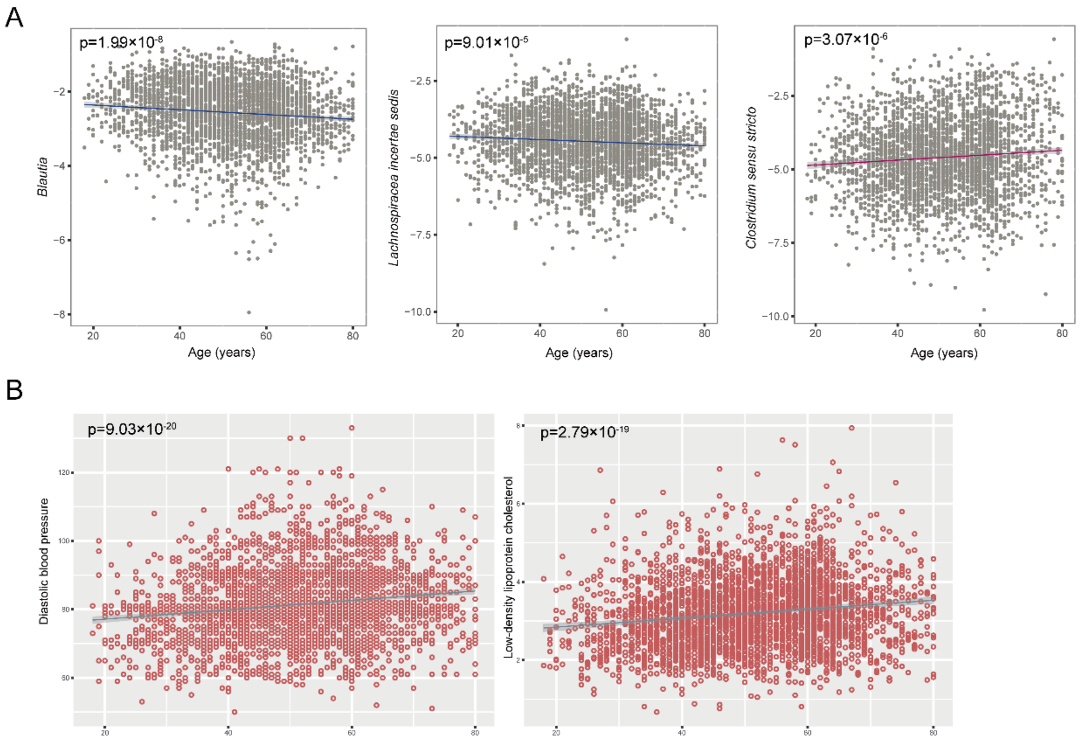


**Figure S12 Associations between genera, clinical indexes and age.** (A) The regression relationship between age-related gut microbiota and age (*n* = 3,224). (B) The tendency of age-related clinical indexes with age ($p < 0.05$).
